# Research on the Relationship between Chinese Elderly Health Status, Social Security, and Depression

**DOI:** 10.3390/ijerph19127496

**Published:** 2022-06-18

**Authors:** Min Shao, Jianwei Chen, Chao Ma

**Affiliations:** 1 School of Public Health, Southeast University, Nanjing 210009, China; shaomini123321@163.com; 2 Institute of Education and Economy Research, University of International Business and Economics, Beijing 100029, China; chenjianwei@uibe.edu.cn

**Keywords:** health status, social security, depression, aging

## Abstract

(1) Background: To explore the relationship between health status, social security status, and the occurrence of depression in older adults and provide a basis for mental health care services for the elderly population; (2) Methods: This study used the 2018 China Health and Senior Care Tracking Survey (CHARLS) data to select 8383 older people aged over 60 years old as the research subjects. The two-category Logistic model was used to analyze the research problem; (3) Results: Older adults with depressive tendencies accounted for 34.1% of the total older adults. The incidence of depression among female older adults reached 41.51%. The risk of depression in the elderly population was 40.3% lower in males than in females (OR = 0.597, 95% CI: 0.539–0.662). Self-rated health status, physical disability, and receipt of pensions affected the incidence of depression in older adults (all *p* < 0.05); (4) Conclusions: Focus on and intervene in the mental status of elderly females and disabled elderly people. Provide mental and economic support and mental health care services to the elderly at the family and social levels. Promote the healthy development of the mental health of the elderly and promote active aging.

## 1. Introduction

With the rapid development of society and the economy, mental health issues have gradually become the focus of public health [1]. The increasing aging of China is making the mental health problems of the elderly become increasingly prominent [2]. According to China’s seventh census, there are 264.02 million people aged 60 and above, accounting for 18.70% of the total population, making it the country with the most significant number of older adults in the world today [3]. At present, 12 provinces have already entered the stage of deep aging. It is predicted that, during the 14th Five-Year Plan period, China’s elderly population will exceed 300 million people and will move from light aging to moderate aging [4].

In the context of such a problematic population aging situation, the rapid increase in the aging population will not just cause economic and care burdens on the family and society [5]. The more severe consequences of the backward construction of elderly care institutions and the absence of family care for the elderly are that the elderly have significant mental health problems, but have not received attention and timely psychological intervention [6]. According to the White Paper on Mental Health of Chinese Urban Residents, two-thirds of Chinese people are in a state of mental sub-health [7], and one-tenth of people have different degrees of mental problems. Mental health problems are more severe in the elderly.

Depression is the most common mental health problem that affects the health of the elderly. Not only will it have a severe impact on the health of the elderly, but it will also reduce the social function and quality of life of the elderly [8]. It will also affect the cognitive function of the elderly and increase the risk of cardiovascular and cerebrovascular diseases [9]. When depression persists, it also increases the risk of suicide and death among older adults [10]. Studies have shown that suicide levels among older adults are higher in rural areas than in urban areas, suggesting inequalities in mental health and resource allocation between rural and urban areas [11]. Therefore, paying attention to depression in older adults and analyzing it can help provide timely psychological interventions and guidance to older adults, which is vital to promote an active aging society [12].

## 2. Materials and Methods

### 2.1. Data Source

**Figure 1:**
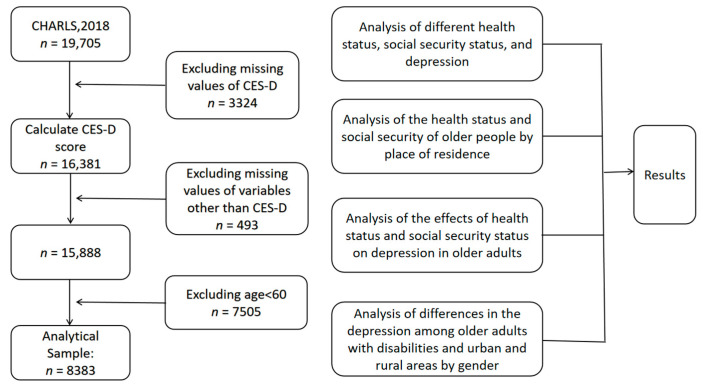
Sample selection, exclusion criteria, and tests performed for various parameters.

The data used in the study came from the 2018 China Health and Retirement Longitudinal Study (CHARLS) [13]. The survey covered 150 counties (districts) and 450 communities (villages) in 28 provinces (autonomous regions and municipalities) across the country. The sample covered 12,400 households and 23,000 respondents [14]. The sample was representative. The research object of this paper was elderly people aged over 60 years old. After cleaning and screening the data, 8383 valid samples were finally retained. The sample selection, exclusion criteria, and some parametric testing steps are briefly shown in Figure 1.

### 2.2. Research Content

The main content of this study is the influence of health status and social security status on the occurrence of depression in older people. The health status included the self-evaluated health status [15], physical health, and chronic disease prevalence. The self-rated health status was divided into five levels, including “very bad”, “bad”, “fair”, “good”, and “very good”, expressed as integers from 1 to 5, respectively [16]. Physical health selected whether there was physical disability as a representative. Chronic disease prevalence selected three representative chronic diseases: hypertension, dyslipidemia, and diabetes. Social security was studied from two perspectives of medical insurance and pension insurance. Medical insurance was based on whether there were one or more types of medical insurance as the standard. No medical insurance was recorded as 0, and one or more types of medical insurance was recorded as 1. The pension insurance situation was based on whether or not they received a pension as the standard. No pension was assigned a value of 0, and in receipt of a pension was assigned a value of 1.

Depression in the elderly was the explained variable in this study, calculated from the Cognitive and Depression Part Depression Scale (CES-D) in the CHARLS questionnaire. The summary table contained eight negative questions and two positive questions. Refer to the existing literature for the treatment method of the depression scale summary table. The number of days in the past week that have been negative was “less than one day” and “1 to 2 days”. The “3 to 4 days” and “5 to 7 days” options were assigned as integers between 0 and 3, and the answer to the positive question adopted the opposite assignment method to the negative question. The total score of CES-D ranged from 0 to 30 points [17]. This study classified depression scores. When the score was less than or equal to 10 points, the sample was considered to not be in good psychological condition. When the score was greater than 10 points, the population was deemed to have a depressive tendency.

### 2.3. Statistical Methods

This study used STATA version 16.0 (StataCorp LLC, Lakeway, TX, USA), and SPSS version 25.0 (IBM, Armonk, NY, USA) for analysis. The χ^2^ test was used to preliminarily analyze depression in the elderly. Logistic regression analysis was performed on health status, social security, and other factors that may affect the mental health of the elderly, and the test level was α = 0.05.

## 3. Results

### 3.1. Analysis of the Essential Characteristics of the Elderly with Different Depression Statuses

Among the samples in this study, women accounted for 49.03%, and men accounted for 50.97%. Among them, the incidence of depression in women reached 41.51%, and the incidence of depression in men was 27.36%. According to this study, the incidence of depression in the elderly population of women was much higher. The difference was statistically significant (*p* < 0.05). Respondents aged 60–70, 70–80, and over 80 accounted for 64.83%, 30.25%, and 4.92% of the total sample. The difference in the incidence of depression in each age group was not statistically significant. The proportion of older people living alone without a partner accounted for 18.63%, the incidence of depression in this population was as high as 43.47%, and the difference was statistically significant.

The incidence of depression was higher in the elderly with hypertension, dyslipidemia, and diabetes, at 38.6%, 40.7%, and 40.6%, respectively. There were statistically significant differences in the occurrence of depression between self-rated health, physical disability, high blood pressure, dyslipidemia, diabetes, medical insurance status, and pension status, as shown in Table 1.

### 3.2. Analysis of the Health Status and Social Security of the Elderly in Different Places of Residence

There were statistically significant differences in mental health, self-evaluated physical health, and social security among the elderly in urban, urban–rural fringe, and rural areas (*p* < 0.05). Rural elderly people are more likely to experience depression than those in urban and urban–rural areas. The number of depressed elderly people in rural areas accounted for 37.51% of the total rural elderly population, much higher than the 23.97% and 29.29% in urban and urban–rural areas, respectively. In terms of medical insurance ownership, whether in urban or rural areas, there was still a small part of the elderly population who did not have any medical insurance. The rate of rural elderly people without medical insurance was even higher, at 2.91%. From the perspective of pension insurance receipt, the vast majority of the elderly in rural areas did not receive pensions. The rate was as high as 92%, as shown in Table 2.

### 3.3. The Impact of Health Status and Social Security Status on Depression in the Elderly

Taking the tendency of depression in the elderly as the dependent variable (No = 0 and Yes = 1), the variables representing the health status, social security status, and other control variables were included in the multivariate logistic model for research. The results of the study, as reported in Table 3, showed that the self-assessed health status, physical disability, and receipt of pensions had an impact on the occurrence of depression in the elderly (all *p* < 0.05). After controlling for individual characteristics, the risk of depression in the elderly with a self-rated health status was 47.2% lower than that of the elderly with very poor health status (OR = 0.528, 95% CI: 0.430–0.655). The risk of depression in the elderly with physical disabilities was 61.5% higher than that of the able-bodied population (OR = 1.615, 95% CI: 1.227–2.125). People who received pension insurance had a 39.2% lower risk of depression than those without pensions (OR = 0.608, 95% CI: 0.529–0.699). It was not found that a single chronic disease and medical insurance impacted the occurrence of depression. This finding is different from previous ones.

In addition to the above key research factors that impacted depression in the elderly, gender being male, living with a partner, having a wage income, and undertaking social activities were protective factors for depression (*p* < 0.05). Males had a 40.3% lower risk of depression than females (OR = 0.597 and 95% CI: 0.539–0.662). The risk in those living with a partner was 30.1% lower than that in those living alone without a partner (OR = 0.699, 95% CI: 0.615–0.795). Older people with an income other than pensions had a 22.3% lower risk of depression than those without an income (OR = 0.777, 95% CI: 0.665–0.907). Participating in social activities also reduced the risk of depression by 14.9% (OR = 0.851, 95% CI: 0.769–0.940).

### 3.4. Comparison of Depression among Older Persons by Gender

A more detailed analysis of men and women’s different mental health conditions was conducted. The results were shown in Table 4. The results found that women with physical disabilities had significantly higher rates of depression than women without physical disabilities. This result also applied to the male population. The depression rate for women with disabilities was 63.48% compared with 44.12% for men with disabilities, with a statistically significant difference (*p* < 0.001).

As shown in Table 5, the highest rate of depressive symptoms was found among women living in rural areas, 45.81%, while the lowest rate of depressive symptoms was found among men in urban areas, 18.63%. The prevalence of depressive symptoms gradually increased from urban to rural areas for men and women. Most rural women had significantly higher depressive symptoms than urban women (*p* < 0.001).

## 4. Discussion

**Figure 2:**
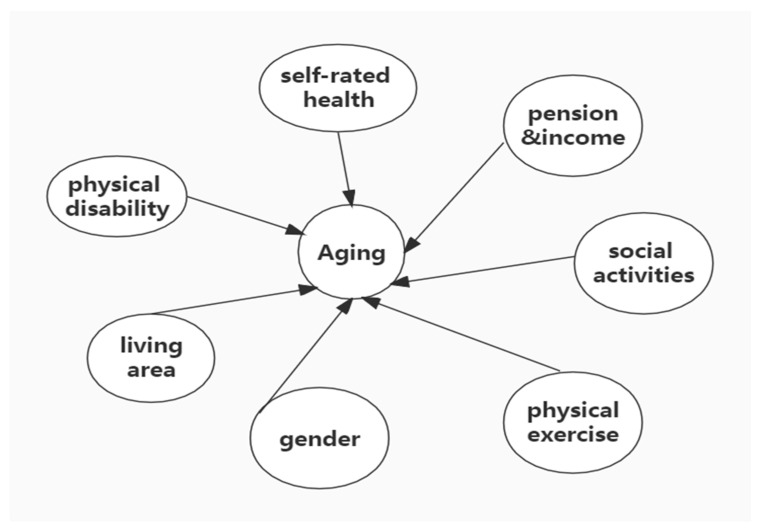
Major factors affecting the mental health of older adults.

Research on the mental health of the elderly in China has shown that factors such as self-rated health status, the number of chronic diseases, living style, intergenerational support [18], gender differences, and urban–rural differences impact the mental health of the elderly. On this basis, this research expands the health status, including self-rated health, common chronic diseases, and physical disability factors. Social security factors include medical insurance and pension insurance [19], making the research content more comprehensive. The final parameters of the findings that affect the health of older adults are shown in Figure 2.

The results show that female elderly people are more likely to be depressed than male older adults. The incidence of female depression in the sample of this study was as high as 41.51%, which is consistent with previous research results [20]. From the psychological differences between men and women, women can be more sensitive than men [21], resulting in women being more susceptible to adverse emotions. From the perspective of the different social environments that men and women face, women will take on domestic work in the family and face more discrimination in the workplace. From the different ways of venting negative emotions for men and women, men can relieve their stress by smoking and drinking alcohol, while most women choose to hold back their emotions [22]. These differences result in women being more susceptible to depression than men [23]. A further study of men and women affected by depression found that women with and without disabilities always suffered from depression at a higher rate than men [24].

This study also found that the incidence of depression in rural elderly people was higher than that in urban and urban–rural areas. The findings also showed that depression was higher among women in rural and combined urban–rural areas than in men; this is generally consistent with the previous findings. This article speculates that the lack of entertainment and exercise infrastructure in rural areas makes it impossible for rural elderly to relieve depression through entertainment and exercise. Some studies have shown that physical activity has an important impact on mental health. A meta-analysis found that exercise for more extended periods was beneficial for improving poor mood [25]. A cross-sectional study by British scholars showed that a decrease in the time spent exercising would significantly increase the probability of mental health problems [26]. For older people with declining physical health, moderate exercise can improve physical fitness and thus reduce the likelihood of depression. The medical and old-age insurance coverage rates for the rural elderly were lower than those in urban areas, making rural elderly people spend more on health to resist disease risks.

In the analysis of health status, social security status, and the depressive factors of the elderly, it was found that good physical condition is key to improving the mental health of the elderly [27]. The self-rated health status had a significant impact on depression. The better the self-rated health of the elderly, the lower the likelihood of depression. Unlike previous studies, this study introduced physical disability factors to study their impact on the mental health of the elderly, and found that physical disabilities will increase the risk of depression in the elderly [28]. Compared with ordinary older people who are disabled due to disability, they need family support and care, and need professional long-term care services [29]. Receiving pensions can effectively reduce the risk of depression in the elderly [30], and, at the same time, the elderly can obtain wages other than pensions, which also promotes mental health. In addition, this study also found that the risk of depression in the elderly without partners and living alone was 30.1% higher than that of the elderly with a partner and living together.

Researchers generally agree that chronic illness affects depression. Some studies have also shown that chronic pain contributes to depression [31]. However, this study has not found evidence that chronic disease significantly influences the onset of depression. The reason may be speculated as follows. With the implementation of China’s grassroots public health policies, chronic diseases were effectively controlled early on, preventing the further aggravation of chronic diseases. In addition, there are active medical insurance policies to help patients with chronic diseases reduce the cost burden [32]. Therefore, compared with other factors, chronic diseases exist for a long time and are effectively controlled, and their degree of influence on depression in the elderly is gradually decreasing.

## 5. Challenges

The increasing aging of society has led to a great deal of social concern for older adults’ physical and mental health. However, there are still many challenges in improving the mental health of older adults. From the perspective of older adults, many older adults have a severe lack of knowledge about mental illnesses due to traditional concepts, which prevents them from promptly detecting that they are suffering from mental illnesses [33]. Therefore, they cannot seek timely psychological counseling help from family members or social workers [34]. From the perspective of the family environment, Chinese older adults may also face problems such as low family support and short time spent with their children [35]. These problems further contribute to the lack of timely help for older adults dealing with psychological problems. The number of older people is very high in society and the country. The current government-constructed geriatric care system and infrastructure planning are still inadequate, and the promotion of the mental health of the elderly still faces tremendous challenges [36].

## 6. Conclusions

In conclusion, considering the many challenges faced by older adults in improving their mental health, this study provides some rationale for implementing a mental health care program for older adults. It can help family members and community workers to implement timely psychological interventions for older adults suffering from depression. It is essential to change the perceptions of older adults themselves, to raise their awareness of mental illness [37], understand its harmful effects, and proactively seek various kinds of help. At the same time, it is also necessary to give full play to the power of society and government to jointly cope with the challenges brought by aging.

Improving the mental health of the elderly and reducing the incidence of depression in the elderly can start with family intervention and social support [38]. From a family perspective, family members must provide the elderly with the necessary emotional support and financial support [39], and timely psychological counseling for depression in the elderly. From the social level, the community should strengthen the care and attention provided to the elderly living alone and provide services such as regular home care for the disabled elderly with physical disabilities [40]. Government departments should protect the elderly in rural areas, such as old-age care and medical issues, provide basic living security for low-income elderly in rural areas, and gradually establish an “Elderly-centered” long-term care system [41].

## Figures and Tables

**Table 1:** Analysis of different health statuses, social security statuses, and depression (*n* = 8383).

Variables	Total	Healthy	Depressive	$\chi^{2}$	*p*
	*n* (%)	*n* (%)	*n* (%)		
Age				3.363	0.186
60–70	5435 (64.83)	3588 (66.02)	1847 (33.98)		
70–80	2536 (30.25)	1637 (64.55)	899 (35.45)		
>80	412 (4.92)	283 (68.69)	129 (31.31)		
Sex				186.165	<0.001
Female	4110 (49.03)	2404 (58.49)	1706 (41.51)		
Male	4273 (50.97)	3104 (72.64)	1169 (27.36)		
Marriage				71.705	<0.001
NO	1562 (18.63)	883 (56.53)	679 (43.47)		
YES	6821 (81.37)	4625 (67.81)	2196 (32.19)		
Self-rated health				904.043	<0.001
Very bad	880 (10.50)	758 (86.10)	122 (13.90)		
Bad	957 (11.40)	775 (81.00)	182 (19.00)		
Fair	4137 (49.30)	2939 (71.00)	1198 (29.00)		
Good	1853 (22.10)	858 (46.30)	995 (53.70)		
Very good	556 (6.60)	178 (32.00)	378 (68.00)		
Physical disability				40.121	<0.001
NO	8132 (97.00)	5390 (66.30)	2742 (33.70)		
YES	251 (3.00)	118 (47.00)	133 (53.00)		
Hypertension				9.031	0.003
NO	7417 (88.50)	4915 (66.30)	2502 (33.70)		
YES	966 (11.20)	593 (61.40)	373 (38.60)		
Dyslipidemia				16.978	<0.001
NO	7548 (90.00)	5013 (66.40)	2535 (33.60)		
YES	835 (10.00)	495 (59.20)	340 (40.70)		
Diabetes				9.3789	0.002
NO	7883 (94.00)	5211 (66.10)	2672 (33.90)		
YES	500 (6.00)	297 (59.40)	203 (40.60)		
Medical insurance				5.971	0.015
NO	211 (2.50)	122 (57.80)	89 (42.20)		
YES	8172 (97.50)	5386 (65.90)	2786 (34.10)		
Pension				193.5306	<0.001
NO	6488 (77.40)	4010 (61.80)	2478 (38.20)		
YES	1895 (22.60)	1498 (79.10)	397 (20.90)		

**Table 2:** The health status and social security of the elderly in different places of residence (*n* = 8383).

Variables	Urban Area *n* (%)	Urban–Rural Area *n* (%)	Rural Area *n* (%)	$\chi^{2}$	*p*
Depressive				112.481	<0.001
NO	1253 (76.03)	396 (70.71)	3859 (62.49)		
YES	395 (23.97)	164 (29.29)	2316 (37.51)		
Self-rated health				77.115	<0.001
Very bad	174 (10.56)	52 (9.29)	654 (10.59)		
Bad	235 (14.26)	76 (13.57)	646 (10.46)		
Fair	887 (53.82)	292 (52.14)	2958 (47.90)		
Good	269 (16.32)	101 (10.04)	1483 (24.02)		
Very good	83 (5.04)	39 (6.96)	434 (7.03)		
Medical insurance				15.580	<0.001
NO	21 (1.27)	10 (1.79)	180 (1.91)		
YES	1627 (98.73)	550 (98.21)	5995 (97.09)		
Pension				10.883	<0.001
NO	521 (31.61)	286 (51.07)	5681 (92.00)		
YES	1127 (68.39)	274 (48.93)	494 (8.00)		

**Table 3:** Logistic regression analysis of health status, social security, and depression (*n* = 8383).

Variables	B	S.E.	Wald	*p*	OR	95% CI
Self-rated health						
Very bad	−2.497	0.137	331.029	<0.001	0.082	0.063–0.108
Bad	−2.088	0.127	270.396	<0.001	0.124	0.097–0.159
Fair	−1.590	0.101	248.784	<0.001	0.204	0.167–0.249
Good	−0.638	0.105	36.808	<0.001	0.528	0.430–0.655
Physical disability						
YES	0.479	0.140	11.723	0.001	1.615	1.227–2.125
Hypertension						
YES	0.111	0.077	2.045	0.153	1.117	0.960–1.300
Dyslipidemia						
YES	0.136	0.083	2.668	0.102	1.146	0.973–1.350
Diabetes						
YES	0.030	0.105	0.079	0.778	1.030	0.838–1.266
Medical insurance						
YES	−0.177	0.154	1.335	0.248	0.837	0.620–1.132
Pension						
YES	−0.497	0.071	48.607	<0.001	0.608	0.529–0.699
Age						
70–80	0.211	0.124	2.882	0.090	1.234	0.968–1.574
>80	0.167	0.125	1.786	0.181	1.182	0.925–1.511
Sex						
Male	0.516	0.053	96.172	<0.001	0.597	0.539–0.662
Marriage						
YES	−0.358	0.065	30.103	<0.001	0.699	0.615–0.795
Education level						
Medium level	0.481	0.376	1.633	0.201	1.617	0.774–3.381
High level	0.181	0.383	0.222	0.637	1.198	0.565–2.539
Income						
YES	−0.253	0.079	10.150	0.001	0.777	0.665–0.907
Social activities						
YES	−0.162	0.051	9.993	0.002	0.851	0.769–0.940
Strenuous exercise						
YES	0.302	0.058	26.893	<0.001	1.352	1.206–1.515
Moderate exercise						
YES	0.014	0.052	0.071	0.789	1.014	0.915–1.124
Light exercise						
YES	−0.072	0.066	1.170	0.279	0.931	0.818–1.060

**Table 4:** Comparison of depression among older adults with disabilities by gender.

Variables	Female (*n* = 4110)			Male (*n* = 4273)		
	*n*	*n*	Rate (%)	*n*	*n*	Rate (%)
Disability						
NO	3995	1633	40.88	4137	1109	26.81
YES	115	73	63.48	136	60	44.12
$\chi^{2}$			23.52			19.85
*p*			<0.001			<0.001

**Table 5:** Comparison of depression among older people by gender in urban and rural areas.

Variables	Female (*n* = 4110)			Male (*n* = 4273)		
	*n*	*n*	Rate (%)	*n*	*n*	Rate (%)
Area						
Urban area	859	248	28.87	789	147	18.63
Urban–rural area	278	96	34.53	282	68	24.11
Rural area	2973	1362	45.81	3202	954	29.79
$\chi^{2}$			84.76			41.29
*p*			<0.001			<0.001

## Data Availability

The data provided in this study can be obtained from the Peking University China Health and Retirement Longitudinal Study database upon request, http://charls.pku.edu.cn/index/en.html (accessed 30 October 2021).
